# Primary extramedullary spinal melanoma mimicking spinal meningioma: A case report and literature review

**DOI:** 10.3892/ol.2014.2099

**Published:** 2014-04-28

**Authors:** YU-PING LI, HENG-ZHU ZHANG, LEI SHE, XIAO-DONG WANG, LUN DONG, ENXI XU, XING-DONG WANG

**Affiliations:** 1 Department of Neurosurgery, Clinical Medical College of Yangzhou University, Yangzhou, Jiangsu 225001, P.R. China; 2 Department of Neurosurgery, Zhenjiang First People’s Hospital, Zhenjiang, Jiangsu 212002, P.R. China

**Keywords:** primary spinal melanoma, malignant melanoma

## Abstract

Primary spinal melanoma is a rare lesion, which occurs throughout the cranial and spinal regions, however, is primarily observed in the middle or lower thoracic spine. The clinical features of primary spinal melanoma are complex and unspecific, resulting in a high misdiagnosis rate. In the present case report, a rare case of spinal melanoma exhibiting the dural tail sign and mimicking spinal meningioma is reported. The initial diagnosis, using magnetic resonance imaging (MRI), was unclear. Thus, melanin-containing tumors and spinal meningioma should have been considered in the differential diagnosis. The tumor was completely resected using a standard posterior midline approach, which was followed by chemotherapy. Subsequent to the surgery, the patient was discharged with improved motor capacity and a follow-up MRI scan showed no recurrence after six months. The present study demonstrates that it is critical for neurosurgeons to focus on increasing the accuracy of initial diagnoses in order to make informed decisions regarding the requirement for surgical resection. The present case report presents the clinical, radiological and pathological features of primary extramedullary spinal melanoma mimicking spinal meningioma to emphasize the importance of early identification and diagnosis.

## Introduction

Melanoma is a highly malignant type of tumor, with a median survival rate of between six and 12 months, and a five-year survival rate of <10% (1). Primary malignant melanoma of the central nervous system (CNS) accounts for 1% of all cases of melanoma. Primary spinal melanoma is particularly rare and may be intra- or extradural, or may possess intra- and extramural components (2). Spinal melanoma is primarily found in the middle or lower thoracic spine. Since the first case of spinal melanoma was reported by Hirschberg in 1906, <100 cases have been reported (3). There are no evidence-based guidelines regarding primary spinal melanoma, for example specific incidence, treatment or prognosis guidelines. The current report presents a case of primary spinal melanoma of the thoracic spine, which presented unusual radiographic features at the time of diagnosis. Magnetic resonance imaging (MRI) revealed that the lesion exhibited typical signs of spinal melanoma and enhanced MRI revealed a dural tail sign. Subsequent to total resection, a six-month follow-up MRI scan demonstrated no tumor recurrence. In the present case, the diagnosis of primary spinal malignant melanoma was obtained through histological, radiological and immunohistochemical analyses. The aim of the present case report is to discuss the diagnosis, treatment and prognosis of this rare condition.

## Case report

**Figure 1:**
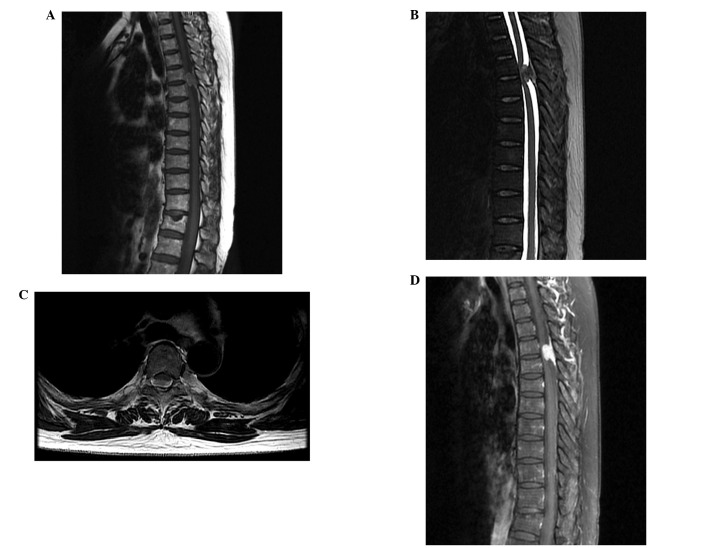
(A–C) Preoperative magnetic resonance imaging showing characteristics of a T4–T5 intradural extramedullary spinal mass. (A) T1-weighted image showing the hyperintense signal lesion. (B and C) T2-weighted images showing a hyperintense signal on (B) sagittal and (C) transaxial images. (D) T1-weighted image with contrast medium showing homogeneous enhancement of the lesion with the dural tail sign.

A 57-year-old female was admitted to the Clinical Medical College of Yangzhou University (Northern Jiangsu People’s Hospital, Yangzhou, China) in December 2012 with a history of bilateral lower-extremity numbness and back pain for one month. One week prior to admission, the patient noticed a more marked tingling sensation and progressive weakness in the lower extremities. Upon admission to hospital, a neurological examination revealed hypoesthesia below the T8 level and progressive weakness (grade IV) of the bilateral lower extremity. The deep-tendon reflexes of the lower extremity were weak and accompanied by uroschesis. The Babinski sign was positive on the two sides. MRI scans of the thoracic spine revealed a space-occupying mass (size, 17×10 mm) with an obvious dural tail sign at the T4–T5 level (Fig. 1). The margins of the mass were indistinct and the adjacent dural exhibited local incrassation and compression of the spinal cord. The lesion was initially diagnosed as a complex spinal meningioma and surgery was performed on the patient.

In December 2012 the patient underwent a T4–T5 laminectomy using a standard posterior thoracic midline approach. The dural mater, which was black in color, was observed at the T4–5 region due to an intradural underlying mass. Upon opening the dura, a black arachnoid that was covered in pigmentation was observed. A vertical incision was made in the arachnoid and a black, oval-shaped, hypervascular mass was found, which measured 15×12 mm. The mass was indistinct, and was strongly adhered to the dura and the arachnoid. The tumor compressed, although did not invade, the spinal cord and was removed completely using a microsurgical technique. Surgery indicated that the tumor was likely to be malignant; therefore, a radical resection was performed on the black-colored dura and arachnoid.

Standard histopathological examination of the tumor samples (hematoxylin and eosin staining; magnification, ×100) revealed cytologic atypia, mitotic activity and tumor cells with cytoplasmic deposition. These cellular characteristics were highly indicative of malignant melanoma. Immunohistochemically, the neoplastic cells stained positive for S-100 protein and the malignant melanoma monoclonal antibody, human melanoma black (HMB) 45. However, the neoplastic cells were negative for epithelial membrane antigen (EMA) and neuron-specific enolase (NSE). The proliferation rate, based on Ki-67 expression, was observed to be high (10%). Thus, the histopathological and immunohistochemical findings indicate that the tumor originated from a melanocyte. Postoperative analyses, including clinical examination, full body computed tomography, MRI, abdominal ultrasound and ocular examination, revealed no lesions in the patient’s other organs.

**Figure 2:**
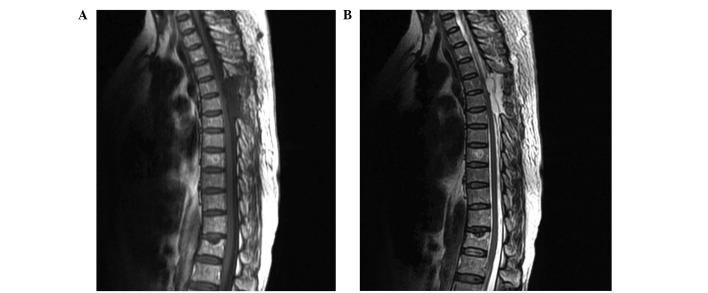
Postoperative (A) T1- and (B) T2-weighted magnetic resonance imaging (MRI) scans. The postoperative MRI scan, performed one week after surgery, demonstrates the complete resection of the mass.

A postoperative clinical examination of the patient revealed no loss of motor capacity or decrease in motor strength. Repeated MRI scans of the thoracic spine, conducted one week after surgery, demonstrated that the mass had been totally resected (Fig. 2). The patient was transferred to the oncology department for chemotherapy, and was followed up by the medical oncology and neurosurgery departments.

The present study was approved by the Ethics Committee of the Clinical Medical College of Yangzhou University (Yangzhou, China) and informed consent was obtained from the patient.

## Discussion

Primary spinal melanoma commonly arises from melanoblasts along the neural crest and typically occurs in the leptomeninges. More than 90% of spinal melanomas metastasize and grow rapidly, which usually results in a fatal outcome within six months (4). Due to the relative rarity of primary spinal melanoma, at present, only 60 cases have been identified in English studies (Table I). The mean age at presentation is 50 years (range, 15–80 years) and thoracic melanomas are the most common type. Among all of the published cases of primary spinal melanoma, including the present case, the patients have presented non-specific and progressive symptoms of myelopathy. These symptoms mimic those of other intraspinal mass lesions, which occupy similar locations and demonstrate similar growth patterns, including spinal meningioma, meningeal melanocytomas and metastatic melanoma.

With regard to radiological examination, MRI scans are commonly used to identify different spinal lesions. The typical pattern of spinal melanoma observed using MRI, includes signal hyperintensity on T1-weighted images and signal isointensity or hypointensity on T2-weighted images. These signal characteristics are inconsistent as the MRI signal depends on the presence of melanin, intratumoral hemorrhages and fat deposits, which complicates the majority of spinal melanoma images. MRI scanning aids diagnosis, however, it does not specifically differentiate between primary melanoma and other malignant lesions. The signal characteristics of MRI may easily lead to an erroneous diagnosis. It is important for surgeons to make an accurate diagnosis and be aware of the limitations of the diagnostic value of MRI. In the present case report, enhanced MRI revealed an obvious dural tail sign, which is a classic characteristic of meningioma. However, T1-weighted images with hyperintensity and T2-weighted images with hypointensity are typical features for melanoma, and atypical for meningioma. Therefore, it is difficult to exclude the diagnosis of spinal meningioma prior to surgery, as intratumoral bleeding may result in an uneven hyperintensive signal in T1-weighted images. In the present case report, the final diagnosis of the patient required further investigation using methods other than MRI.

Histologically, melanin-containing tumors, including melanocytosis, melanocytoma, malignant melanoma and meningeal melanomatosis, exhibit spindle or epithelioid cells arranged in sheets, bundles, nests or whorls containing variable quantities of melanin pigment in the cytoplasm (5). Accurate pathological diagnoses are important as the histological distinction, clinical course and prognosis vary for different melanin-containing tumors. Furthermore, appropriate case-specific therapy, involving surgery, and radio- and chemotherapy should be planned on the basis of a specific diagnosis. The differential diagnosis between malignant melanoma and meningeal melanocytoma require consideration, as the two originate from melanocytes. In the present case report, the presence of the histological characteristics of tumor necrosis, cytologic atypia and high mitotic activity resulted in the initial diagnosis of a malignant melanoma. Therefore, distinguishing between malignant melanoma and melanotic meningioma or metastatic carcinoma is important. Immunohistochemical analysis facilitates the differentiation between these different melanin-containing tumors. Positive staining of the anti-melanoma antibody, HMB45 and the S-100 protein indicates that cells are of melanocytic origin. A negative reaction for EMA eliminates the possibility of a mass being a melanotic meningioma of the spinal cord, and a negative reaction for EMA and NSE exclude metastatic carcinoma of melanocytic origin. Thus, in order to accurately diagnose primary spinal malignant melanoma, it is important to combine histological, radiological and immunohistochemical analyses.

In the present case, complete surgical resection was recommended in order to obtain a curative outcome. Local control rates have been reported to be four-fold higher if complete resection is achieved (6). Intraoperatively, the differentiation between various melanin-containing tumors is often difficult. Certain typical features, including dura mater attachment, an indistinct mass and a dark, black color may indicate that the tumor has originated from leptomeningeal melanocytes. Spinal meningioma may mimic this appearance, when the lesion presents within a large volume of hematoma. Therefore, pathological and immunohistochemical analyses of the resected specimen are required to provide a specific diagnosis. The selection of an appropriate individual therapy, for example radio- and/or chemotherapy, is based on all of these findings. A previous study reported that Gamma Knife therapy improved the clinical outcome and reduced the complication rate in metastatic CNS melanoma (7). However, the efficiency and long-term survival rate of Gamma Knife therapy requires further investigation to confirm these findings. Despite treatment strategies involving total resection and adjuvant therapy, the prognosis of patients with primary spinal melanoma remains particularly poor. Therefore, close follow-up studies are required, even in cases of complete surgical resection.

The efficacy of radio- and chemotherapy remains controversial in the treatment of melanoma. While melanoma is a radiotherapy-resistant tumor, patients benefit from surgical resection, which has been reported to significantly alleviate the symptoms that result from its compressive effect. Surgical resection has also been reported to reduce the growth rate of melanoma (60). Furthermore, Hamilton *et al* (61) reported preoperative radiotherapy in a patient with spinal melanoma and obtained satisfactory clinical outcomes. The radiotherapy dose depended on the tumor size, location, compression symptoms and patient tolerance; however, a dose of 12–24 Gy was recommended by the majority of the doctors. Attitudes towards adjuvant treatment vary worldwide. A study in the USA reported that high-dose interferon treatment improved patient prognosis, although it resulted in severe side-effects (62). A meta-analysis showed that chemo- and biological therapy were capable of reducing the recurrence rate and increasing survival by only 3% after five years (63). Previous studies have shown that treatment with chemotherapy and/or novel monoclonal antibodies, for example using ipilimumab, overcomes cytotoxic T-lymphocyte antigen 4-mediated T cell suppression and improves overall survival (64,65). The patient in the present case was treated with chemotherapy subsequent to surgery and no tumor recurrence was observed at the six-month follow-up.

In conclusion, the clinical features of primary spinal melanoma are complex and may be easily misdiagnosed as other spinal lesions. In the current report, a case of primary malignant melanoma of the thoracic spine is presented. Primary malignant melanoma is a particularly rare and aggressive tumor, therefore, total resection is recommended. An accurate diagnosis based on histological and immunohistochemical analyses of the resected tissue, is critical for selecting the appropriate therapy to enhance patient outcome. Unlike the majority of cases of primary intradural melanoma, the present case exhibited unusual radiological features, including a dural tail sign that mimicked a spinal meningioma. Thus, the present case report illustrates the importance for neurosurgeons to analyze radiological data carefully to increase the accuracy of their initial diagnosis. The diagnostic potential of malignant melanoma requires consideration at the time of surgery to establish the need for aggressive surgical resection. Thus, early complete surgical resection followed by individualized radio- or chemotherapy may enhance patient outcome. Furthermore, a meta-analysis focuses on the best treatment strategy for this disease and aids with the diagnosis and treatment of primary spinal melanoma.

## Figures and Tables

**Table I:** Summary of the 60 cases of primary spinal cord melanoma reported in English studies.

Case	First author (ref)	Year	Location	Age (years) /Gender	Laminectomy	Adjuvant treatement	Metastasis	Survival duration (months)	Condition of follow-up end point	Duration symptom (months)
1	Yu (8)	2012	C2–C6	48/M	Y	Y	N	2	Alive	6
2	Yan (9)	2012	L2–L4	44/F	Y	N	N	NR	NR	24
3	Fuld (10)	2011	C2	62/M	Y	Y	N	11	Alive	NR
4	Vij (11)	2010	C1–C2	40/M	Y	N	N	12	Alive	9
5	Lee (12)	2010	C1–C6	39/M	Y	Y	N	14	Alive	11
6	Kolasa (13)	2010	T10	57/F	Y	Y	N	12	Alive	2
7	Kim (14)	2010	T4	34/F	Y	N	N	36	Alive	12
8	Kwang (15)	2010	T7–T8	68/F	Y	Y	N	6	Alive	
9	Kounin (16)	2005	C2–C4	41/F	Y	N	N	3	Alive	9
10	Kwon (17)	2004	C6–C7	45/F	Y	Y	N	8	Alive	48
11	Tosaka (18)	2001	CSF	20/M	N	NR	Brainstem; leptomeningeal	5	Succumbed	7
12	Farrokh (19)	2001	T12-L1	80/F	Y	N	N	9	Alive	NR
13	Bidzinski (20)	2000	C6–C7	36/M	Y	Y	N	48	Alive	8
14	Brat (21)	1999	T10	71/F	Y	N	N	14	Alive	NR
15			C1	52/M	Y	Y	N	16	Alive	NR
16			C4	20/F	Y	N	N	20	Alive	NR
17			C4	57/F	Y	Y	N	8	Succumbed	NR
18	Salame (22)	1998	T9–T10	76/F	Y	Y	N	15	Alive	6
19	Francois (23)	1998	T8	62/M	Y	N	NR	28	Alive	18
20	Salpietro (24)	1998	C3	62/M	Y	Y	Brain	15	Succumbed	1
21	Magni (25)	1996	T	64/M	Y	Y	N	18	Alive	24
22	Yamasaki (26)	1989	T7–T8	31/M	Y	Y	N	23	Alive	6
23	Schneider (27)	1987	L3–L4	68/F	Y	N	Cerebellum; frontal	10	Alive	NR
24	Larson (28)	1987	T6–T8	73/M	Y	Y	Leptomeningeal dissemination	84	Alive	6
25			T9	63/M	Y	Y	N	156	Succumbed	96
26			T9–T11	67/F	Y	Y	N	Short period	Alive	18
27			C1–C3	57/F	Y	Y	N	30	Succumbed	3
28			T9–T10	69/F	Y	N	N	45	Succumbed	24
29	Ozden (29)	1984	T7–T10	30/F	Y	Y	N	16	Alive	NR
30			C1–C6	15/F	Y	N	N	18	Alive	NR
31	Holaday (30)	1968	S2	20/F	Y	N	NR	12	Succumbed	3
32	Clifford (31)	1968	C3–C5	64/M	Y	N	N	24	Succumbed	4
33	Kiel (32)	1961	C4–C6	33/F	Y	NR	Cerebral; leptomeningeal	19	Succumbed	25
35	Hirano (33)	1960	T	42/F	Y	NR	NR	NR	Alive	1
36	Zimmerman (34)	1958	D9–D10	42/M	Y	N	N	4	Succumbed	8
37	Gibson (35)	1957	T	51/F	N	Y	Leptomeningeal dissemination	NR	Alive	NR
38	De Roca (36)	1954	T	50/F	Y	Y	NR	NR	Alive	6
39	Perino (37)	1953	T	40/M	Y	Y	NR	NR	Alive	NR
40	King (38)	1952	L	53/M	Y	N	Dura mater; base brain	NR	Alive	12
41	De Assis (39)	1951	L	26/M	Y	Y	NR	NR	Succumbed	7
42	King (40)	1951	L	47/M	Y	N	Brain; leptomeningeal	NR	NR	2
43	Forbes (41)	1950	T	57/M	Y	N	Leptomeningeal dissemination	NR	Alive	NR
44	Kissel (42)	1950	C	25/F	Y	N	NR	NR	NR	2
45	Castaner (43)	1950	L	52/F	Y	NR	NR	NR	NR	12
46	Mackay (44)	1942	C	32/F	N	NR	Cervical, spinal cord	NR	Alive	10
47	Garcin (45)	1941	L	52/M	Y	Y	NR	NR	NR	3
48	DaCosta (46)	1939	T	55/F	Y	N	NR	NR	NR	24
49	Schnitker (47)	1938	D9–D10	49/F	Y	Y	Lung, liver, uterus, leptomeningeal	6	Succumbed	30
50	Van Bogaert (48)	1933	T 6	38/M	Y	NR	NR	NR	NR	6
51	De Blasi (49)	1930	T	71/F	Y	NR	NR	NR	Alive	6
52	Bell (50)	1930	C	48/F	Y	NR	NR	NR	NR	3
53	Prussak (51)	1929	T	29/M	Y	NR	NR	NR	NR	3
54	Ringertz (52)	1926	T	61/F	Y	NR	Brain not examined	NR	NR	6
55	Schmid (53)	1926	T	71/M	N	NR	Cerebral, dura mater	NR	NR	14
56	Koelichen (54)	1916	T	25/M	Y	NR	NR	NR	NR	1.5
57	Lindbom (55)	1912	C1–C3	45/F	N	NR	NR	NR	NR	2
58	Kawashima (56)	1910	C	26/F	N	NR	Leptomeningeal dissemination	NR	NR	7
59	Esser (57)	1907	T1–T2	32/M	Y	NR	NR	NR	NR	0.5
60	Boit (58)	1907	T8–T11	51/M	N	NR	Liver, spleen	NR	NR	11
61	Hirschberg (59)	1906	T	67/F	N	NR	NR	NR	NR	3

C, cervical; S, sacral; T, thoracic; L, lumbar; Y, yes; N, no; NR, not reported; M, male; F, female.
